# Compound food failure on a deteriorated mast system drives Japan’s bear conflict crisis

**DOI:** 10.1126/sciadv.aej8134

**Published:** 2026-09-23

**Authors:** Hengjun Xiao, Zhengwang Zhang, Yoshiaki Miyamoto, Tomohiro Ichinose

**Affiliations:** ^1^ Graduate School of Media and Governance, Keio University Shonan Fujisawa Campus, Fujisawa, Kanagawa 252-0882, Japan.; ^2^ MOE Key Laboratory for Biodiversity Science and Ecological Engineering, College of Life Sciences, Beijing Normal University, Beijing 100875, China.

## Abstract

Climate change is recognized as a key amplifier of human-wildlife conflict, yet the underlying mechanism remains poorly understood. Japan’s 2025 bear crisis, which produced 232 casualties, has been widely attributed to rapid population growth, but demographic processes are far too gradual to explain the surge. Using an 18-year dataset across five Tohoku prefectures, we find that it reflects bears responding to a rhythm breakdown of the forest’s food supply. Conflict rises sharply once mast production falls below a threshold. Around 2006, the mast system passed a break point, after which lean years grew more severe and crossed that threshold more often. Conflict is further amplified when the alternative food source, indexed by primary productivity, fails alongside mast, and the 2023/2025 outbreaks provide a natural experiment separating compound from single-source failure. Beyond the Japanese bear-mast system, our analysis yields a generalized diagnostic framework that considers failing food rhythms, compound shortages, and the thresholds at which animals enter human space.

## INTRODUCTION

**Fig. 1.**
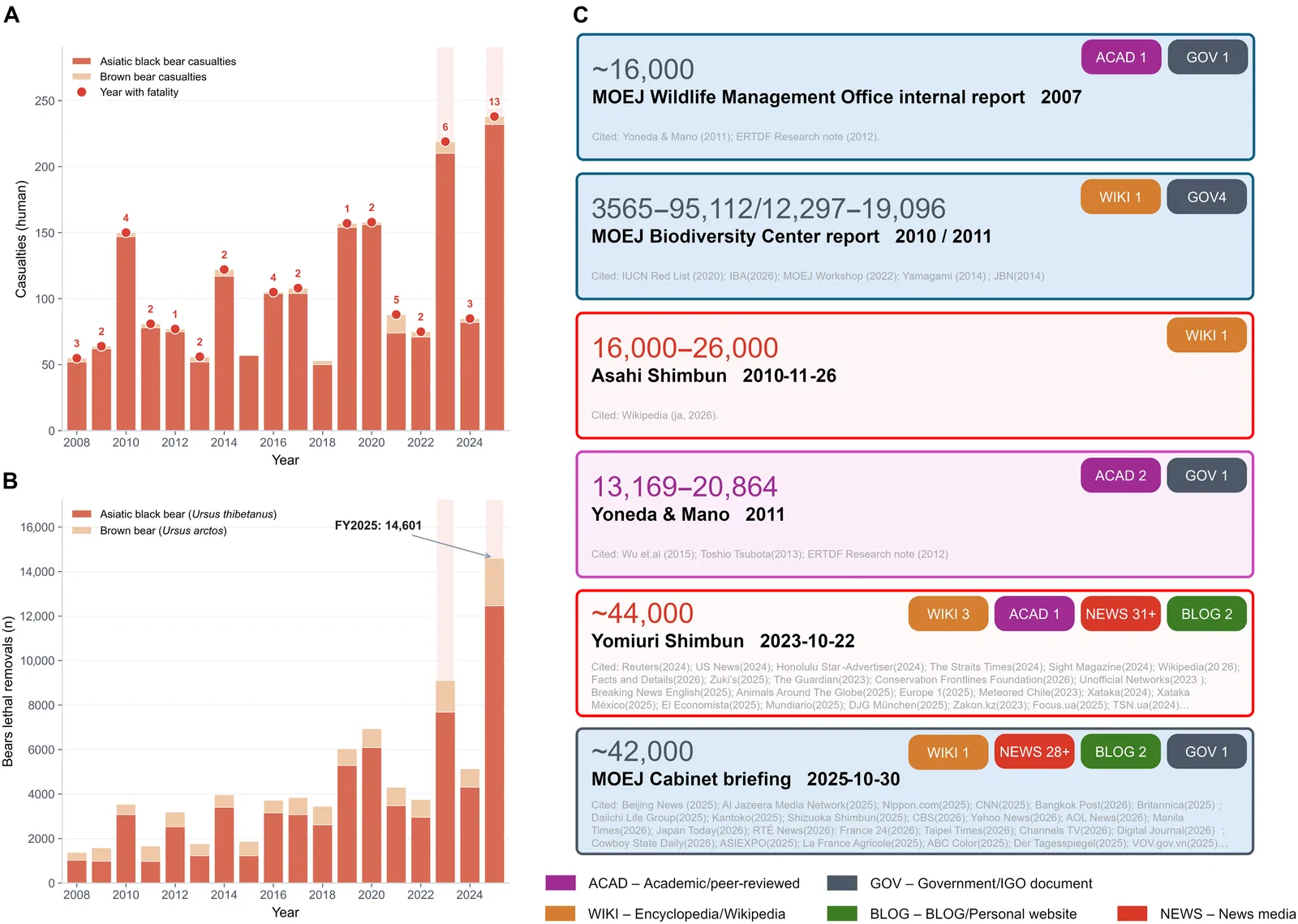
Long-term context for the bear conflict and the population estimate citation pathway. (**A**) Annual human casualties caused by bears in Japan, fiscal years 2008–2025. Red points and numbers of mark years with reported fatalities. (**B**) Annual lethal removals of bears over the same period. (**C**) Citation and reuse history of major published or publicly circulated estimates of the Japanese Asiatic black bear population. Each row represents an estimate family, with source, year, and downstream reuse summarized; colored chips indicate the type and number of downstream citations or reproductions. Full list of cited sources is available in the Supplementary Materials.

Human-bear conflicts across Japan have recently escalated to unprecedented levels. In 2025 alone, the Japanese populations of Asiatic black bears *Ursus thibetanus japonicus* caused a total of 232 human casualties, including 11 fatalities (Fig. 1A) (*1*), representing the highest annual toll on record. The sharp rise in bear-inflicted injuries has prompted the Japanese government to roll out unprecedented mitigation measures, including the deployment of Japan Self-Defense Forces personnel to assist municipal hunters in November 2025 (*2*) and the lethal removal of more than 14,000 bears through nuisance permits (*1*).

One prevailing explanation invokes rapid population growth (*3*), with a widely cited newspaper analysis claiming bear numbers have tripled over the past decade (*4*). However, 65,514 Asiatic black bears have been lethally removed since 2008, averaging more than 3000 per year even excluding the 2025 surge (Fig. 1B) (*1*). Set against the only peer-reviewed population estimate available for this period (*5*), this amounts to removing 15 to 24% of the population each year. That rate approaches or exceeds the maximum intrinsic growth rate documented for *Ursus* species, achieved by *Ursus americanus* only under full protection in optimal habitat well below carrying capacity (∼20%/year) (*6*). Sustaining the population at the current estimate of more than 42,000 against this harvest record is therefore demographically implausible (Fig. 1C). Conflict incidents continued rising through this period, reaching an all-time high in 2025, suggesting that additional ecological factors contribute to the observed pattern.

Beyond population growth explanations, food is the most extensively documented driver of bear-human conflict. Asiatic black bears in Honshu rely heavily on hard mast for prehibernation hyperphagia (*7*). When mast is insufficient, they cannot build enough reserves and must range far more widely for alternative calorie sources to survive the coming winter (*8*). The abundant and high-value food around human settlements, such as garbage and crops, becomes a strong attractant in these lean years, accordingly increasing the frequency of encounters with people and driving nuisance bear incidents at the prefecture scale in Tohoku (*9*). But settlements are dangerous as well as rewarding. Individuals that venture into them are killed in large numbers (*10*), and this mortality reduces overall survival in years of heavy urban use (*11*). Bears therefore normally avoid settlements (*12*) and enter them primarily when natural food becomes severely scarce, suggesting a behavioral threshold in their use of human space.

As global climate change continues, growing evidence indicates that hard mast production is undergoing substantial change. Masting in *Fagus* is triggered by temperature cues (*13*) that normally align flowering with the tree’s stored resources (*14*, *15*). Warming appears to erode this alignment, disrupting the classic rhythm of lean and bumper years. In a warmer scenario, trees may differentiate flower buds even in resource-poor years they would normally skip, raising reproductive effort and drawing down stored nitrogen faster than it is replenished (*16*). The formed flowers thus may be lost due to lack of resources before they mature into seed (*17*). As warm summers become common, the cue is met more often and trees may also respond to it less sharply, so flowering grows less synchronous within the local forest (*18*). A tree flowering out of step with its neighbors receives little pollen, and this loss of efficiency can feed back to desynchronize seed maturation further (*19*).

This dampening of interannual variation and synchrony is already visible in European beech (*Fagus sylvatica*) (*18*), most acutely at cold range margins (*20*). The similar signal has also been documented in Tohoku’s *Quercus crispula*, which has shifted toward shorter masting cycles (*21*). Since masting governs the temporal rhythm of food across entire ecosystems, its breakdown rewrites the foraging landscape for hard mast–dependent consumers. Meanwhile, this disruption can not only act in isolation but also combine with other climatic stresses. For instance, a mast failure year that also comes with a deficit in alternative foods would leave bears with little to fall back on. Our earlier commentary described exactly this for the spring of 2025 (*22*), when anomalous cloudiness cut regional primary productivity by nearly a quarter and likely reduced the young foliage and invertebrates that bears rely on. Such compounding of climate stresses is itself becoming more frequent (*23*), and climate change is also recognized as a formal amplifier of human-wildlife conflict (*24*), including for bears in Japan (*25*). Despite a substantial literature, however, the specific ecological pathway by which climatic stress translates into conflict outbreaks remains underexplored (*26*).

Given the complex interplay of drivers behind bear-human conflict, any explanation resting on a single factor is bound to leave blind spots. We therefore propose a framework of compound food failure and examine three major components together. First, we ask what behavioral condition turns food shortage into conflict, and whether conflict climbs steeply once mast falls below a critical level. Second, we ask whether the mast rhythm itself has shifted. Because mast production here follows a classic cycle of lean and bumper years, we track how each phase has changed over time and trace the reproductive chain to identify where any shift arises. Third, we test how mast and alternative foods compound, asking whether shortage arriving in the same year drives more conflict than either alone. We apply this framework to an 18-year prefecture-year dataset from the five Tohoku prefectures, the country’s highest-density bear range and the epicenter of the crisis, using forestry flower–mast records, MODIS photosynthesis dataset, and incident statistics. We also estimate how much of the recent surge is attributable to the changes we identified and find that it reflects a forest ecosystem in transition, with direct implications for management.

## RESULTS

### Relationship between food supply and bear attack

**Fig. 2.**
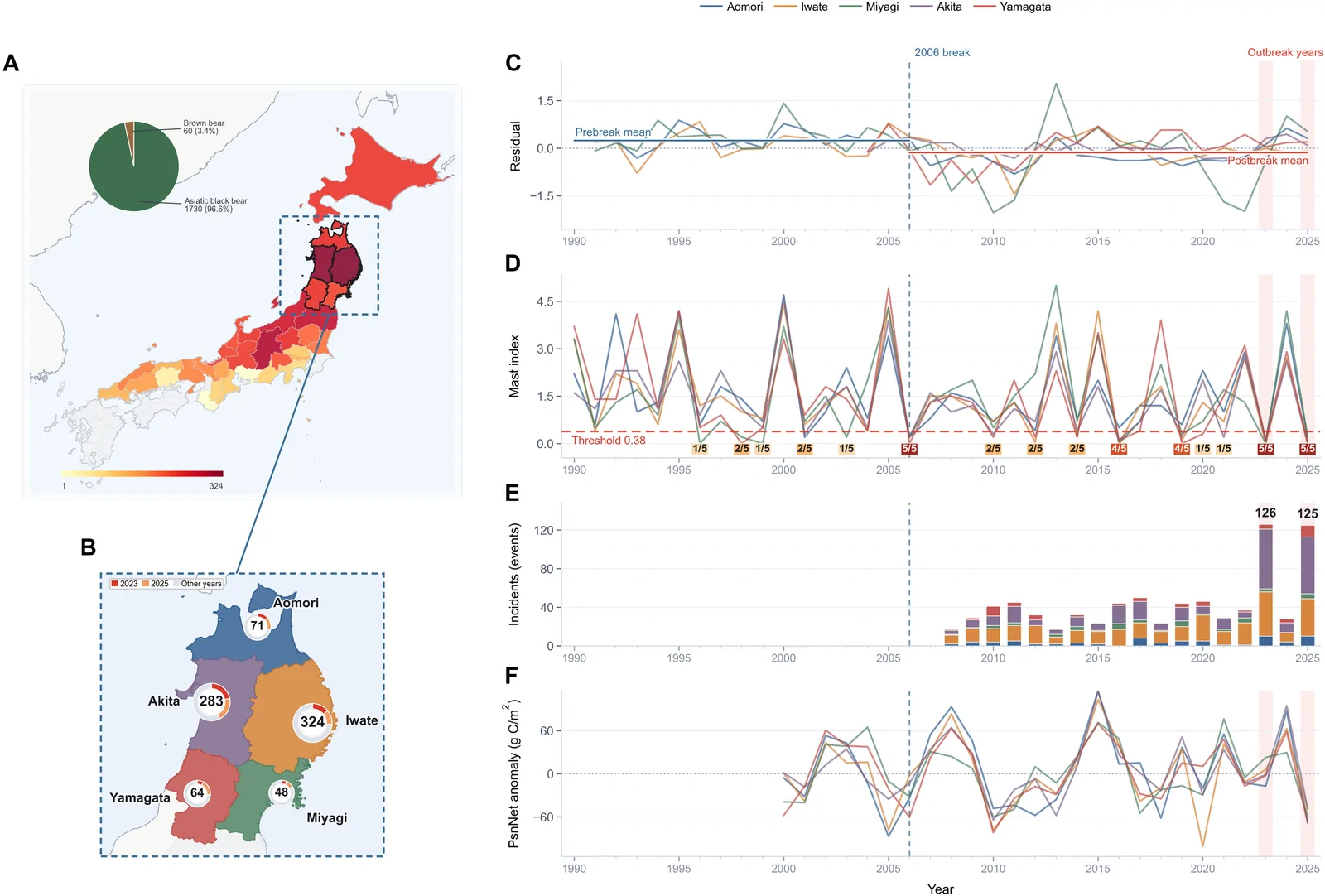
Coupled ecological and conflict indicators across the five Tohoku prefectures. (**A**) Japan-wide context for cumulative bear attack incidents (injury or fatality) since 2008, colored on a logarithmic scale. The inserted pie chart summarizes the species’ composition. (**B**) Situation of five study prefectures in Tohoku. Donut markers show cumulative prefecture-level incidents, partitioned into 2023, 2025, and all other years. (**C**) Annual conversion efficiency residuals from flower to mast production. Horizontal reference segments indicate the pre- and postbreak means, with the estimated regime shift year separating the two periods. (**D**) Prefecture-level MI through time; the dashed horizontal line marks 0.38 threshold (MI_t_). Bottom labels indicate years in which at least one of the five prefectures fell below the threshold. (**E**) Annual bear incidents count stacked by prefectures. (**F**) Annual detrended net photosynthesis anomaly (PsnNet_d_; grams of carbon per square meter) by prefecture.

We first examined the incident record and considered whether it reflected a simple upward trend. Of the 790 incidents recorded in the five Tohoku prefectures across 2008–2025, 251 (32%) fell in 2023 and 2025 alone (Fig. 2, D and E). When yearly incident counts were fitted against time alone using a negative binomial generalized linear mixed-effects model (NB-GLMM), conflict exhibited a significant upward trend [β = +0.061, *P* < 0.001, incidence rate ratio (IRR) = 1.062], seemingly consistent with a sustained ∼6%/year rise. As we show below, however, this signal is neither linear nor stable over time.

**Fig. 3.**
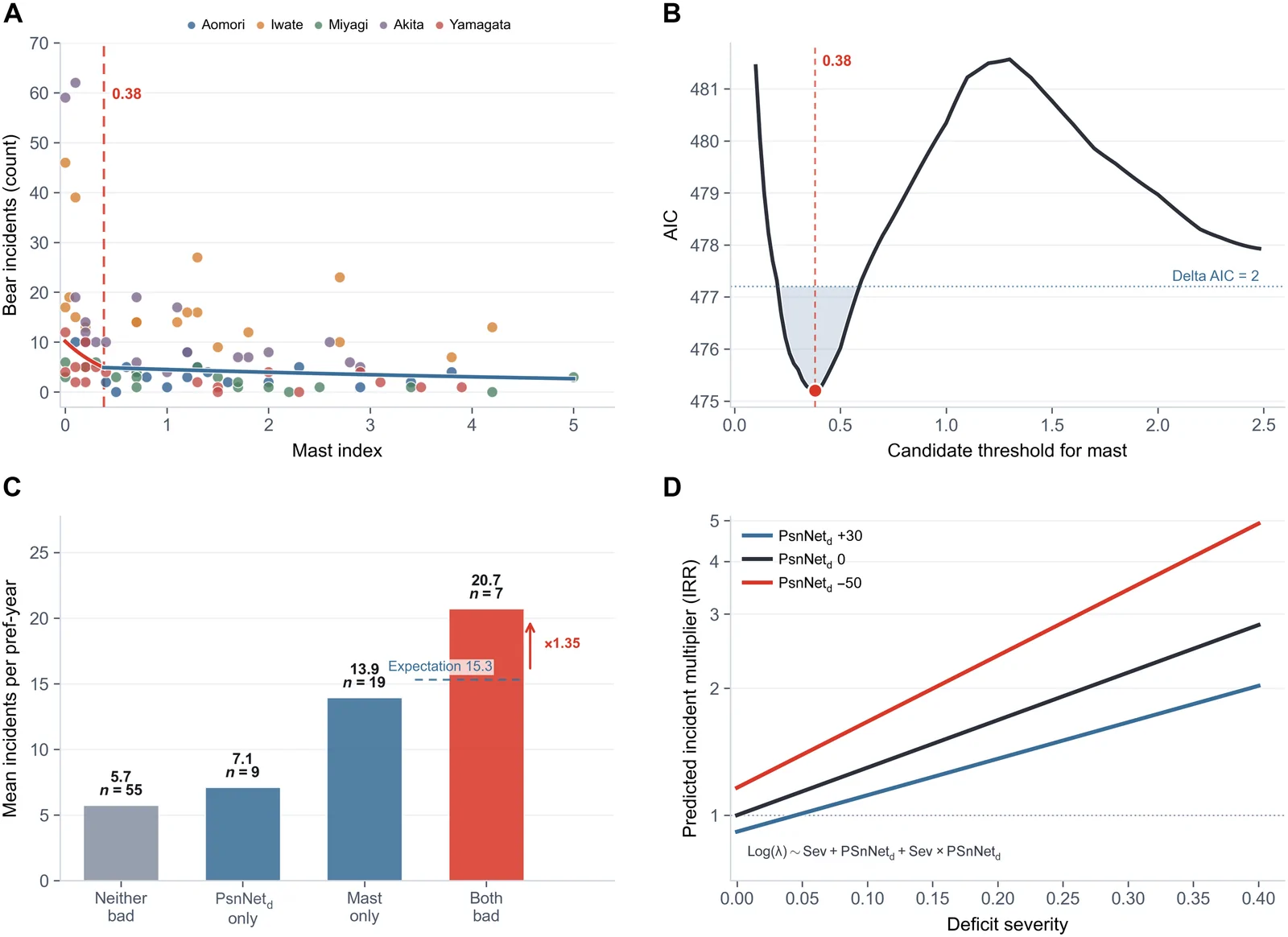
Threshold and dual-deficit structure linking ecological stress to bear incidents. (**A**) Relationship between MI and prefecture-year bear incidents across the five study prefectures. Points show observed prefecture-year values, colored by prefecture; the vertical dashed line marks the selected mast failure threshold. (**B**) Threshold sensitivity profile from candidate MI thresholds in the incident model. (**C**) Mean incident counts per prefecture-year under four ecological states: neither bad, detrended net photosynthesis (PsnNet_d_) deficit only, mast failure only, and simultaneous dual deficit. The dashed horizontal segment shows the additive expectation, and the arrow marks the observed excess above that expectation. (**D**) Model-implied interaction surface for mast deficit severity (Sev) and PsnNet_d_ anomaly, expressed as predicted incident rate ratios.

When mast availability was considered, we found that it does predict the count of incidents, but the relationship was sharply nonlinear (Fig. 3A). Fitting a generalized additive model (GAM) with thin-plate smooth on mast index (MI) yielded an effective degree of freedom (EDF) of 2.27 (*P* < 0.001), and piecewise NB-GLMM grid search then located the threshold at MI = 0.38 (AIC = 475.2), denoted as MI_t_. Below this threshold, incidents climb sharply (β = +1.92, *P* < 0.001), 14.8-fold steeper than the slope above it (β = −0.13, *P* = 0.054). The threshold was identified as a band between MI 0.22 and 0.58, within which the fit was indistinguishable (ΔAIC < 2; Fig. 3B). Crisis years (MI < 0.38) averaged 15.8 incidents per prefecture-year against 5.9 in safe years, and for the rest of the analysis, we use the mast deficit severity (Sev) = max(0, 0.38 − MI) as the below-threshold shortage metric.

We also identified a second food channel that acts alongside mast. Detrended net photosynthesis (PsnNet_d_), which is an index of growing season productivity and a proxy for alternative food supply bears rely on when mast is scarce, predicts conflict independently (β = −0.004, *P* < 0.001), with higher productivity linked to fewer incidents. The 2 × 2 dual-deficit decomposition shows that the two shortages amplify each other: Prefecture-years with neither food channel bad averaged 5.7 incidents; mast-bad alone averaged 13.9; PsnNet_d_-bad alone averaged 7.1; but joint failure of both channels produced 20.7 incidents, well above the ∼15 expected if the effects merely summed (Fig. 3C). A drop in productivity does little on its own but sharply worsens a year in which mast has already failed (Fig. 3D).

### The structural breakdown in 2006

**Fig. 4.**
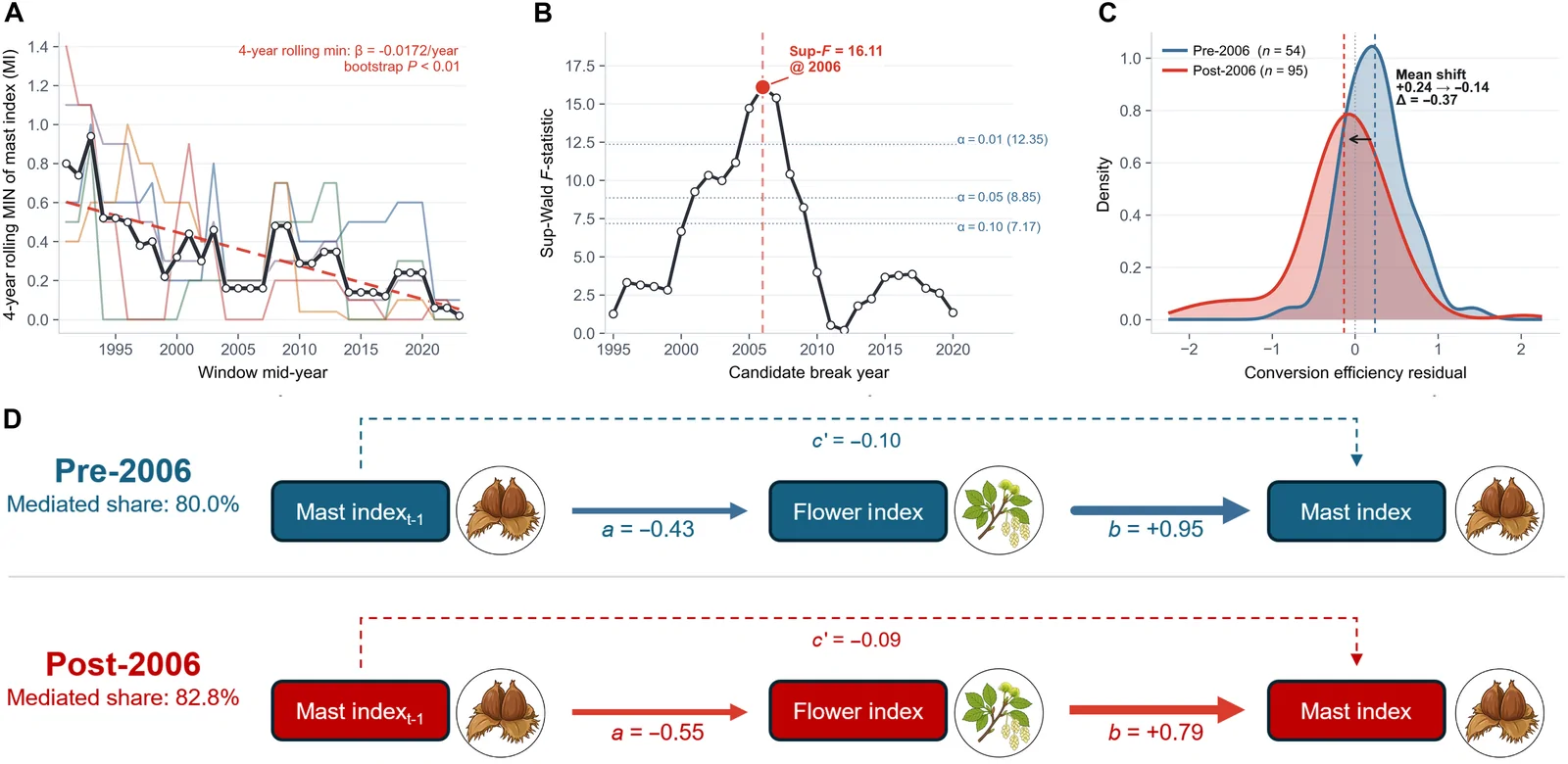
Increasing mast system fragility and a postbreak shift in conversion efficiency. (**A**) Four-year rolling minimum of MI for each study prefecture, with the cross-prefecture mean shown as a highlighted series. (**B**) Sup-Wald structural break profile for candidate break years in the conversion efficiency residual series. (**C**) Kernel density distributions of conversion efficiency residuals before and after the selected break year. (**D**) Serial mediation schematic linking lagged mast, flowering, and current mast before and after the break. Solid arrows represent the indirect pathway, and dashed bottom arrows show the direct residual pathway *c*′.

Across 1990–2025, mast production only exhibits a marginal decreasing trend (*P* = 0.050), but this average hides an asymmetrical change, as the lean years and bumper years move differently. Within successive 4-year windows, the leanest years (the floor of the mast system) grow significantly worse, while the bumper years (the ceiling) show no clear trend (Fig. 4A and table S1). Notably, the significant decline is specific to mast. The lowest values of MI have fallen faster than the lowest of flower index (FI) (*P* < 0.001). Low-mast events have therefore become more frequent, with the annual probability of mast dropping below the crisis threshold MI_t_ rising significantly (logit-GLMM odds ratio = 1.066/year, *P* = 0.016).

Along the reproductive chain from flowering to mature seed, three independent tests together located the decline at the flower-to-mast conversion stage. Annual flowering itself shows no time trend (Mann-Kendall and mixed-effects both *P* > 0.5), so trees are not producing fewer flowers. How strongly a bumper mast year limits the next year’s flowering exhibits no significant time trend (*P* = 0.120). Instead, the significant time trend lies in the residuals of mast regressed on that year’s flowering (β = −0.010, *P* = 0.032), indicating that a given amount of flowering now yields progressively less mature seed.

This weakening of the flower-to-mast conversion was confirmed in a fuller model that also accounts for the previous year’s mast and for year-to-year autocorrelation [AR(1)–generalized estimating equations (GEEs) with AR(1) correlation; see Materials and Methods; β = −0.010/year, *P* = 0.004] and is also found to be discrete rather than gradual. A Sup-Wald level shift test localized the abrupt change point at 2006 (Sup-Wald *F* = 16.11, Andrews’ α = 0.01 envelope: 2005–2007; Fig. 4C and table S2), and dynamic programming change point detection picked the same year independently.

The features of the 2006 break suggest a regional-scale common driver behind it. The flower-to-mast conversion residual moves coherently across the five prefectures (mean pairwise correlation *r* = 0.39 over 2004–2025). After 2006, the distribution of these conversion residuals was both worse on average and more variable, with its mean falling from +0.24 to −0.14 and its spread widening 1.6-fold. The same break weakened mast production in two ways simultaneously (Fig. 4D), with a given year’s flowering converting to mast less efficiently and the carry-over penalty from the previous year’s mast deepening (each *P* ≤ 0.001; table S3). Both patterns are ones that local, prefecture-specific causes could not produce.

Mediation analysis decomposed how 1 year’s mast carries over to the subsequent year’s mast, showing that both before and after 2006, this effect remained firmly mediated (∼80%) by flowering (Fig. 4D and table S4). Because the direct path that acts on mast production without going through flowering is minimal, the year-to-year correlation is not an independent channel but a downstream consequence of prior masting regulating current flowering. What broke in 2006 was the slack in each of its two parts, both the upstream MI_lag1_ (previous year’s MI) → FI coupling deepened and the FI → MI conversion weakened.

Because these two shifts offset one another in their product, the average mast level and its boom-bust rhythm changed little. Yet, each part of this flower-to-mast chain independently slipped toward a lower level, pulling the lean-year troughs lower without a corresponding decline in the bumper peaks. This is exactly the asymmetric decline pattern we found, and the remaining questions are quantitative: How much of the post-2008 incident record is attributable to this regime shift, and do the years it generates share a common outbreak signature or split into distinct modes?

### Counterfactual attribution of the 2006 regime shift

Attributing the surge to the 2006 shift requires a proper model to quantify how food shortage causes conflict. Of six candidate NB-GLMM models compared by AIC, the lowest-AIC specification is model *M*_e_ (table S5), which carries a Sev × PsnNet_d_ interaction, consistent with a compound disaster multiplier. In this model, Sev carries by far the strongest effect (IRR = 13.46, 95% CI = [6.03, 30.05], *P* < 0.0001), adding about 30% more incidents for every further 0.1 that mast falls below MI_t_. The main effect of PsnNet_d_ is correspondingly weak (IRR = 0.997, *P* = 0.015, only ×1.35 even for a large 100-unit fall in growing season productivity), but its interaction with Sev magnifies this effect (β = −0.020, *P* = 0.034), raising that same 100-unit drop to roughly 2.3-fold once mast has failed. The panel mean-centered year (Year_c_) enters as a smaller but still significant additive term (IRR = 1.025, *P* = 0.029).

However, the Year_c_ term, which captures the superficial ∼6%/year upward trend in conflict identified previously, is not a stable feature. When 2023 and 2025 were dropped from the model, the mast shortage (Sev) and vegetation productivity (PsnNet_d_) retained their significance, whereas the time term (Year_c_) lost significance (*P* = 0.138). Refitting the model without Year_c_ also left the Sev × PsnNet_d_ interaction more significant (*P* = 0.008 versus *P* = 0.034; ΔAIC for dropping Year_c_ is only +2.48). Thus, the time trend signal was carried entirely by the two extreme years, and the ecological mechanism itself is time invariant.

**Fig. 5.**
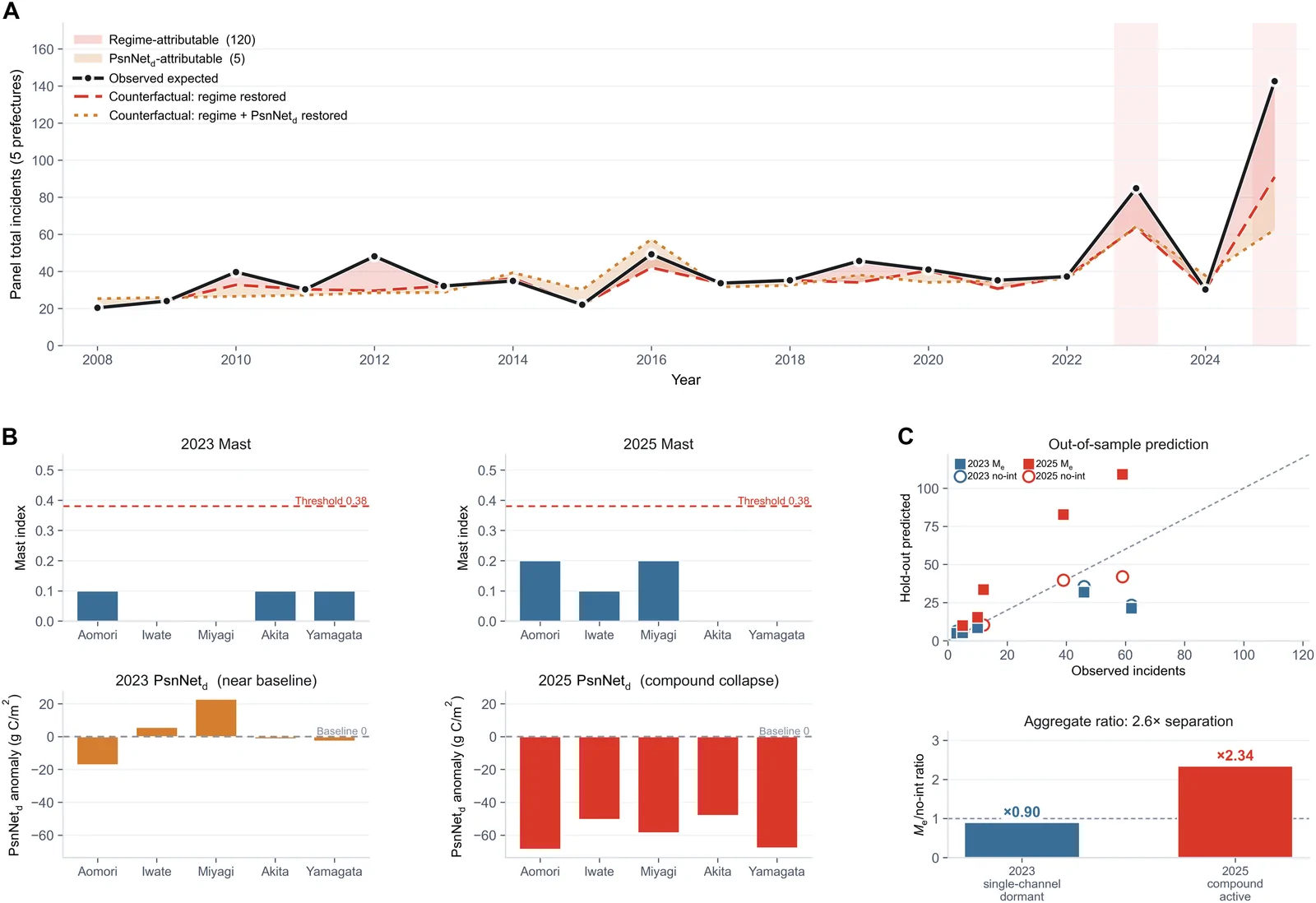
Counterfactual attribution and outbreak typology under the fitted incident model. (**A**) Model-predicted annual incident totals for the five study prefectures under the observed ecological history and two counterfactual scenarios. The black line shows expected incidents from the fitted negative binomial mixed model, red dashed line shows the counterfactual prediction after restoring postbreak mast deficit severity (Sev) to the prebreak regime using the rank-preserving *z*-score mapping, and the orange dashed line further sets detrended net photosynthesis (PsnNet_d_) anomaly to zero. (**B**) Outbreak typology for 2023 and 2025 across the five prefectures. Top compares MI against the mast failure threshold (MI_t_), while bottom compares PsnNet_d_ anomalies against the zero-anomaly baseline. (**C**) Out-of-sample validation of the two-modes-one-model framework. Top: For each outbreak year, prefecture-level observed incidents are compared to predictions from M_e_ and a no interaction comparator, both refitted on data excluding the held-out year. Bottom: aggregate M_e_/no interaction prediction ratio.

Projecting the post-2006 mast production onto the pre-2006 distribution (Fig. 5A) and predicting the resulting incidents with *M*_e_ suggest that restoring the earlier regime could prevent 120 of the 786 expected incidents (15.3%), equivalent to roughly 2.7 years’ worth of baseline incidents (baseline = 43.9 incidents/year × 18 years). At the prefecture-year level, 8 of 26 crisis prefecture-years (31%) would not have crossed MI_t_ under the pre-2006 regime. Restoring PsnNet_d_ alone to baseline prevents only 20 incidents net across the panel (2.5%), but within productivity deficit years, it could prevent 90 (11.5%), mirroring the compound multiplier structure in which PsnNet_d_ matters most precisely when mast has already failed. Jointly restoring both prevents 126 incidents (16.0%); the two food channels do not add because they overlap in the same compound prefecture-years and the NB log link compounds their effects multiplicatively. The attribution is robust, holds at 14.1% on the balanced panel of three prefectures, and stays within 12.2 to 16.1% across the ΔAIC < 2 envelope (table S6).

Consistent with the time-invariant reading established above, 3 years since 2006 (2006, 2023, and 2025) saw all five Tohoku prefectures simultaneously cross MI_t_, but only 2023 and 2025 fall inside the incident analytical window (2008 onward) and can be compared at the outbreak count level. These two years both produced an order of magnitude more incidents than the two near-miss years, 2016 and 2019, in which only four of five prefectures crossed (Fig. 2).

The two 5-of-5 outbreak years of 2023 and 2025 were not the same kind of event, sharing similar mast signatures but completely different primary productivity signatures. The 2023’s mean PsnNet_d_ anomaly was +1.4 (range, −17 to +23), while 2025’s was −58.7 (range, −68 to −48), with every prefecture simultaneously more than one SD below the out-of-sample baseline (Fig. 5B). Classified by this, 2023 is a single-channel outbreak (pure mast failure on a fragile post-2006 baseline), and 2025 is a compound-channel outbreak (mast failure jointly with synchronous regional productivity collapse). Consistent with this split, restoring productivity to baseline in addition to mast prevents no further incidents in 2023 but a further 28 in 2025, exactly as a single-channel versus compound-channel outbreak predicts.

That the same *M*_e_ accommodates both modes is testable out-of-sample. If the Sev × PsnNet_d_ interaction encodes the compound mode, then predicting each outbreak year from the other years should give *M*_e_ and a no interaction comparator nearly identical for 2023 predictions but substantially different for 2025 predictions. As predicted, the ratio of predictions from the interaction model (*M*_e_) to the no interaction model is 0.90-fold in held-out 2023 and 2.34-fold in held-out 2025 (Fig. 5C and table S7). The interaction term itself depends on the contrast between the two outbreak years. It remains significant when either 2023 or 2025 is excluded alone but loses significance when both are removed together (table S8), as expected for a natural experiment in which two contrasting modes jointly anchor the predictor space. The linear interaction recovers the qualitative mode separation cleanly but overextends in absolute magnitude at the most extreme combinations, indicating that the underlying compound response is saturating.

## DISCUSSION

### The current picture of the Japanese black bear

Current demographic evidence on the Japanese black bear is sparse and uneven. The population estimate now used in policy (≈42,000 individuals, 14.5%/year increase rate) (*3*, *27*) derives from harvest-based hierarchical Bayesian models (*28*), the assumptions of which have been questioned on methodological grounds (*29*, *30*). The direct, vital rate evidence points instead to slower reproduction: Placental scar capture-recapture in central Japan (*31*) yields an upper-bound Euler-Lotka growth rate of ≤1.8%/year (text S1), while reproductive tract examination of Honshu females shows late first ovulation and frequent loss of entire litters within the first year (≈27%), with neonate survival as the limiting reproductive step (*32*). Range expansion across Honshu has separately been attributed primarily to agricultural abandonment and reduced snowfall (*33*). The direct evidence is therefore sparse and points in the opposite direction from the policy driving estimate and, in any case, operates on timescales too slow to account for a 2-year crisis.

Behavioral ecology, by contrast, is well characterized. Asiatic black bears depend heavily on hard mast for prehibernation hyperphagia (*7*), and the consequences of mast shortage are documented across multiple axes: Energy intake efficiency drops (*8*), dietary specialization intensifies as each bear resorts to whatever it can find (*34*), movement and exposure to human-dominated areas rise in lean seasons (*35*), bears undertake long-distance forays beyond their natal home range in poor-mast years (*36*), and they progressively concentrate in lowland habitats with more reliable food (*10*). Stable isotope analyses (δ^15^N ≈ 3 to 5 per mil) place this species well below a vertebrate-predator trophic position, with a diet dominated by plants and insects (*37*); even in mast failure years, when sika deer contribution rises (*38*), the increase reflects scavenging rather than predation (*39*). Bear-on-human attacks in Japan are accordingly predominantly defensive rather than predatory (*40*), consistent with hungry animals encountering people in the course of foraging.

If food drives these encounters, then one alternative is that depopulation has improved forest food supply and inflated bear numbers. Yet, rural depopulation has not triggered spontaneous habitat recovery in Japan’s seminatural landscapes (*41*); these secondary forest systems instead face serious degradation pressures across Honshu (*42*, *43*). Tohoku, despite hosting Japan’s most severe human-bear conflict, has one of the country’s lowest farmland abandonment rates (*44*); an abandonment-driven mechanism would moreover predict a gradual decadal trend. Combined with the demographic and behavioral evidence above, rapid deterioration of bears’ food resources in the forest offers the most parsimonious explanation.

### From floor descent to compound failure

Wildlife should not enter human-occupied space as a default behavior. Even apex carnivores treat humans as a primary fear stimulus (*45*), nonlethal human disturbance elicits antipredator response sets equivalent to actual predation (*46*), and the resulting landscape of fear suppresses foraging at community scale (*47*); entering human-occupied space is therefore an expensive decision. Foraging itself, however, is state dependent: Animals tolerate sharply higher risk only after energetic reserves fall below a critical floor (*48*, *49*), formalized as risk-prone foraging under the energy budget rule (*50*, *51*). When bears cross into this high-cost landscape, the marginal value of food must have risen above the perceived encounter cost. The empirical threshold at MI_t_ = 0.38 sits well below the value at which mast is categorically reported as failed (MI ≈ 1.0). This is exactly what a behavioral threshold predicts: Bears tolerate severe shortage and cross into human landscapes only when natural food falls substantially below that failure level.

Unfortunately, our results suggest that bears are crossing this behavioral threshold more frequently because the food system’s floor has descended toward it. Rolling 4-year minima of MI have declined, while maxima held steady (Fig. 4A), meaning that lean years grew leaner without bumper years changing. This is opposite to the pattern documented in European beech, where both percentiles of seed production rise under warming (*52*), indicating that masting breakdown is species and system specific in direction. Mediation analysis localizes the decay to the flower-to-mast conversion and shows both within-year efficiency and the carry-over from the previous year’s mast affected (Fig. 4C). The biological consequence is reproductive investment expended without fitness return. Because flowering in temperate masting trees is regulated by stored nitrogen (*14*), flowers that form but fail to mature represent resources already allocated and now lost to neither seed production nor next cycle recovery (*53*), eroding tree fitness as much as bear food supply.

Mechanistically, the 2006 break is precisely located in time and coincides with a climate regime shift in which the regional summer mean temperature has not fallen below 19°C in any of the 20 years since that time (fig. S1). The discrete climate change closely matches the signature of the conversion failure we have observed, but its proximate physiology is still unresolved. From the flower induction mechanism as now understood (*13*, *54*–*57*), we outline a candidate rectifier failure hypothesis (text S2): A cool summer temperature veto once let trees skip reproduction in low-nitrogen years, acting on the masting rhythm much as a rectifier acts in an electronic circuit; warm summers now eliminate this veto, so the floor collapses while the ceiling holds. However, alternative drivers (pollination failure and environmental veto) cannot yet be ruled out, and future physiological evaluation is needed.

Mast deterioration alone, however, does not exhaust the food supply pathway. PsnNet_d_ indexes the herbaceous biomass (*58*) and arthropod resources (*59*) that bears fall back on when hard mast is poor, and it enters the incident model independently of mast. The two channels compound rather than add at the level of attack events (Fig. 3, C and D). Two nonexclusive mechanisms can produce this: Anthropogenic food in human settlements (garbage, orchards, and livestock feed) is essentially unaffected by natural shocks and so spikes in relative attractiveness during compound deficit years (*11*); and severe nutritional deficit raises bear glucocorticoids and down-regulates risk perception (*60*, *61*), blunting the fear barrier described above. Earlier accounts in which dietary plasticity buffered *Ursus* against mast shortages (*62*) tacitly assumed a fallback substrate that did not itself fail, an assumption no longer met once both channels collapse together.

Treated as a natural experiment, the 2023 and 2025 outbreaks separate the compound mechanism from its single-channel counterpart in a way the within-panel regression cannot. They produced outbreaks of comparable magnitude through ecologically distinct paths (Fig. 5B). The crisis in 2023 was a severe single-channel mast failure on near-baseline PsnNet_d_, the classical bear-mast outbreak (*9*), while the 2025 crisis combined a comparable mast deficit with synchronous PsnNet_d_ collapse. The substantive point is not that the interaction term is real but that mechanistically distinct paths now converge on comparable crisis magnitudes. This is what we expect when the system buffer has descended close enough to the behavioral threshold that either a severe single-channel shock or a moderate compound one suffices to cross it. The pattern is the multivariate compound mode of human-wildlife conflict (*23*, *63*). Forecasting systems that monitor only mast availability will therefore miss compound-channel years entirely while still catching severe single-channel ones.

### Implications for management and the scope of the framework

If the recent surge traces to ecological conditions on a fragile post-2006 baseline, then the appropriate management response also differs. Current management is calibrated to a sustained demographic trend, whereas the data instead support an episodic, ecologically triggered pattern concentrated in two extreme outbreak years; the implied intervention timing differs accordingly. Removal reduces local bear numbers; the ecological drivers identified here operate on independent timescales and are not modified by removal.

Large-scale removal under these conditions is unlikely to have a durable effect, and it carries both demographic and genetic risk. Anthropogenic space use reverses once natural food recovers (*11*), and a meta-analysis of 77 bear intervention cases finds lethal control effectiveness declining linearly to zero by ∼10 years and turning counterproductive thereafter (*64*). Removal under food stress has been documented to produce a 57% female population decline (*65*). Genomic data also reveal low genetic diversity and strong differentiation among already fragmented peripheral populations (*66*), indicating that such removals fall on units with little capacity to absorb them. Such regimes have historical precedents at scale: In 19th century Tasmania, the bounty on the thylacine (*Thylacinus cynocephalus*) was passed without independent verification of the damage estimates put forward by its proponents (*67*); the species went extinct by 1936.

Two operational alternatives use data streams that already exist. First, joint deterioration of the forestry flower/MI and primary production data across prefectures distinguishes compound outbreak risk from tolerated single-channel years, allowing preparedness measures proportionate to mechanism. Second, the carcasses generated by current culling represent an unused demographic data stream: Tooth cementum (*68*) and placental scar (*69*) analysis on culled bears in three central Honshu prefectures already recovers age structure, reproductive parameters, and mortality rates directly (*31*), with an institutional precedent in Sweden, where hunters submit reproductive organs from harvested bears for systematic analysis (*70*). Neither alternative imposes the demographic costs documented above.

There are several scope caveats that should be acknowledged. First, 10 of 90 prefecture-years (2023 and 2025 across all five prefectures) account for roughly a third of the panel’s incidents, making aggregate statistics sensitive to these extremes. Second, the fallback resource proxy PsnNet_d_ (*58*, *59*) establishes that a second food channel does matter but does not identify which resource (herbaceous biomass, arthropods, or soft fruits) operates. Third, the analysis is restricted to Tohoku; whether the MI_t_ = 0.38 threshold and the compound mechanism generalize to bear conflict zones with different climates and forest composition is still left open.

Regardless of which physiological mechanism is ultimately validated, the framework developed here contributes to the broader literature on climate-driven human-wildlife conflict (*24*). The compound food failure decomposition is portable to other mast-dependent *Ursidae* × *Fagaceae* systems where only single-channel monitoring is now used. Each system displays its own masting breakdown signature under warming. European beech shows both percentiles of seed production rising over time (*52*). Tohoku *Fagus* shows a descending floor with a preserved ceiling. What generalizes is therefore not a single directional prediction but the diagnostic framework itself. The framework comprises compound food failure interactions and behavioral threshold responses, both of which single-driver gradient forecasts systematically miss.

## MATERIALS AND METHODS

### Study area and data sources

We selected five Tohoku prefectures located in the northern part of Japan (Aomori, Iwate, Miyagi, Akita, and Yamagata) to conduct our analysis. This region is considered to host the highest-density populations of Asiatic black bears (*5*, *71*) and accounts for the most severe human-bear conflict (*1*). The cool temperate broadleaf forests of this region are overwhelmingly predominated by Japanese beech *Fagus crenata* (*72*). The produced hard mast here is the primary autumn food source for bears (*7*), with strong interannual variability (*73*) in mast production that has been shown to predict subsequent year nuisance removal frequency at the prefecture level (*9*).

Bear injury incident events and lethal removal counts were obtained from the Ministry of the Environment of Japan (MOEJ) Bear Information Hub (*1*), using the dataset as updated on 15 May 2026. For incident modeling, we used the count of incident events per prefecture-year over fiscal years 2008–2025 caused by Asiatic black bears, drawn from the MOEJ injury statistics bulletin. We use event counts not casualty numbers (number of people injured or killed) on methodological grounds: Casualty counts conflate the underlying conflict rate with the number of people present at the moment of an encounter; event counts more cleanly reflect the underlying bear-human attack rate. National lethal removal counts of permit-based bear captures, which, in 2025, represented 99.2% of total captures, were drawn from MOEJ’s preliminary statistics.

Flower and mast data were obtained from the Tohoku Regional Forest Office Buna (beech) flowering and fruiting survey (*74*), using the dataset of 2025 version. The survey conducts annual visual observations at 145 fixed plots distributed across the five study prefectures. Flowering intensity is assessed in early summer and mature seed production in autumn. At each plot, canopy abundance of flowers or seeds is assigned a categorical score (5 across the whole canopy, 3 across the upper canopy only, 1 for very sparse, and 0 for absent), and per-plot scores are averaged within prefecture to give an annual prefecture-year FI and MI on a continuous 0 to 5 scale. By the survey’s own categorical interpretation, prefecture-year MI ≥ 3.5 is judged a bumper year, 2.0 to 3.5 average, 1.0 to 2.0 failure, and <1.0 severe failure. Aomori, Iwate, and Miyagi records span 1990–2025; Akita and Yamagata records span 2004–2025. To preserve mechanistic interpretation of the potential break year, the balanced subset (Aomori, Iwate, and Miyagi) was used for structural break and mechanism probe analyses; the full five-prefecture panel was used for incident modeling.

Beyond hard mast, bears fall back on other food channels when mast is poor, such as understory herbaceous biomass, arthropods, and soft fruits. Lacking direct census data on these diffuse resources, we use remote-sensed productivity, which is widely used as a proxy for forage and resource availability in large-mammal ecology (*75*), on the rationale that the primary productivity of an area shapes its entire food web; the approach has been applied across herbivores and nonherbivores alike, including *Ursus* species (*76*). We therefore indexed this alternative channel with net photosynthesis (PsnNet), derived from the MODIS MOD17A2H Net Photosynthesis product (*77*) and aggregated to prefecture-year totals over the active growing season (2000–2025).

### Modeling the mast-incident relationship

Incident counts were modeled by NB-GLMMs (*78*) with prefecture random intercepts; the NB family was preferred over Poisson on overdispersion (variance/mean = 14.1; ΔAIC = +25.7). Year was centered at the panel mean (Year_c_), and parameter estimates are reported with 95% Wald confidence intervals. Before entering the model as a covariate, prefecture-year PsnNet was standardized within prefecture and LOWESS-detrended (fraction = 0.5, a ∼13-year span chosen to remove only the multidecadal drift while retaining interannual variation), so that the covariate reflects year-specific deviations rather than the long-term productivity background drift; mixed-effects regression confirmed that the detrended PsnNet (PsnNet_d_) has no direct association with MI conditional on the autoregressive masting signal, establishing the two as independent food supply axes.

Nonlinearity in the mast-incident relationship was tested with a GAM (*79*) using a thin-plate spline on Mast (*k* = 5) under an NB family, controlling for Year_c_ and PsnNet_d_; an EDF above 1 indicates departure from linearity. The threshold was located by piecewise NB-GLMM grid search over MI ∈ [0.10, 2.50] in 0.02 increments, selecting the cut-point minimizing AIC; threshold (MI_t_) identifiability is reported as the ΔAIC < 2 envelope. The retained cut-point defines hunger severity as Sev = max(0, MI_t_ − MI), used throughout. A 2 × 2 contingency of mast-bad × PsnNet_d_-bad prefecture-years examined whether joint failure exceeds the additive expectation in mean incident count.

### Detecting the structural break in the forest mast system

The mast deterioration was localized at the flower (FI) to mast (MI) conversion stage on the full 1990–2025 vegetation record. Rolling 3- and 4-year minima and maxima of MI were regressed on year with block bootstrap Mann-Kendall inference (2000 replicates, block length =window). Per-prefecture trends in the MI floor minus FI floor difference were tested with mixed-effects regression. Three further checks ruled out alternative loci: Annual flowering was tested for trend (Mann-Kendall and mixed-effects regression); rhythm stability was tested via FI ∼ MI_lag1_ × Year_c_; and conversion stage decline was tested by inspecting residual time trends from MI ∼ FI.

The flower-to-mast conversion was modeled as MI ∼ FI + MI_lag1_ using AR(1) GEEs (statsmodels 0.14.4) (*80*), the covariance structure motivated by Durbin-Watson diagnostics on prior mixed-effects residuals (positive autocorrelation in four of five prefectures). A break in conversion was searched in two independent ways on the balanced subset (*n* = 105 prefecture-years): (i) a Sup-Wald level shift test (Andrews 1993) on within-prefecture demeaned conversion residuals over 26 candidate years (1995–2020) with 15% trimming, evaluated against Andrews’ size-corrected critical values (*k* = 1, 15% trim; α = 0.10, 0.05, 0.01: 7.17, 8.85, 12.35); and (ii) dynamic programming change point detection (ruptures 1.1.10, L2 cost) (*81*). The biological channel of the break was characterized with a dual-interaction GEE on the same subset [MI ∼ FI × post + MI_lag1_ × post, AR(1) covariance], separating shifts in within-year per-pulse FI → MI efficiency from shifts in interannual reproductive-cost coupling.

The reproductive-cost coupling was further decomposed into a flowering-mediated channel and a direct residual via two-equation mediation, fit separately in each regime on the balanced subset: an *a*-path (FI ∼ MI_lag1_) and a *b*-path (MI ∼ FI + MI_lag1_), both estimated by mixed-effects regression with prefecture random intercepts; the indirect effect was computed as *a* × *b* and the direct residual as *c*′. Within-prefecture moving block bootstrap (block length = 4 years, 1000 replicates) gave 95% confidence intervals on each path coefficient and on the post-minus-pre regime contrast for *a*, *b*, *c*′, *a* × *b*, and the total effect.

### Counterfactual attribution and outbreak mode classification

The final incident model used for attribution was selected by AIC from six candidate NB-GLMM specifications (M_a_ to M_f_) varying inclusion of Year_c_, PsnNet_d_, and their first-order interactions with Sev. The fraction of incident load attributable to the regime shift was estimated by rank-preserving *z*-score mapping. A prebreak mixed-effects conversion model M_pre_ (MI ∼ FI + MI_lag1_, prefecture random intercept) was fit on years through the identified break year. For each postbreak observation *i* in prefecture *k*

$${\text{MI}}_{\text{cf},i}={\hat{f}}_{\text{pre}}\left({\text{FI}}_{i},{\text{MI}}_{\text{lag}1,i}\right)+{\hat{b}}_{\text{pre},k}+\frac{e_{i}}{\sigma_{\text{post}}}\cdot \sigma_{\text{pre}}$$ (1)

where ${\hat{f}}_{\text{pre}}$ is the preregime fixed-effect surface, ${\hat{b}}_{\text{pre},k}$ represents the prefecture random intercept, *e_i_* is the postregime conversion residual, and σ_pre_, σ_post_ is the pre- and postregime residual SDs. The mapping preserves the rank order of weather-driven anomaly within regime while undoing the regime-induced level shift; counterfactual Sev follows as Sev_cf_ = max(0, MI_t_ − MI_cf_).

Two counterfactual scenarios were evaluated under the selected incident model with all random effects included (re.form = NULL): (i) Sev → Sev_cf_ (regime restored only) and (ii) Sev → Sev_cf_ together with PsnNet_d_ → 0 (regime and PsnNet_d_ jointly restored). The attributable fraction is the percentage difference between observed expected total and each counterfactual total. Attribution robustness was checked on the three balanced panel prefectures (Aomori, Iwate, and Miyagi) and at the two endpoints of the ΔAIC < 2 threshold envelope, MI_t_ ∈ {0.22, 0.58}, against the selected MI_t_ = 0.38.

### Outbreak typology and falsification tests

Two mirror falsification tests separated a secular trend interpretation from an episodic outlier interpretation: refitting the selected incident model without Year_c_ to see whether the mechanism terms (Sev, PsnNet_d_, and Sev × PsnNet_d_) retained significance and refitting a Year_c_-including specification on the truncated panel excluding 2023 and 2025 (*n* = 80) to see whether the Year_c_ slope retained significance. Synchrony of postbreak outbreaks was tabulated as the number of prefectures simultaneously crossing the mast threshold per year. Outbreak years (5-of-5 prefecture crossings) were classified as compound channel if the minimum within-year prefecture PsnNet_d_ fell below μ_baseline_ − σ_baseline_ and as single channel otherwise; baseline statistics were computed on prefecture-years excluding 2023 and 2025 so that they cannot influence their own classification.

The two-modes-one-model interpretation was evaluated by leave-one-year-out hold-out prediction across the two outbreak years. For each held-out year ∈ {2023,2025}, M_e_ and an otherwise identical NB-GLMM omitting the Sev × PsnNet_d_ interaction term were refit on the panel excluding *y*; both models were then used to predict prefecture-year incidents in *y*, and the predicted-incident ratio (M_e_/no interaction comparator) was reported per prefecture and aggregated across the five prefectures. Robustness of the Sev × PsnNet_d_ interaction term was further evaluated by refitting M_e_ on three additional truncated panels—excluding 2023 only, excluding 2025 only, and excluding both—and reporting the interaction coefficient, its SE, and the ΔAIC against the no interaction comparator on each panel.

### Usage of generative AI

During this work, Claude Opus 4.8 (Anthropic) was used to assist with English language editing, and the GitHub Copilot extension (GitHub/Microsoft) in Visual Studio Code was used for code autocompletion and refactoring during development of the analysis code. Neither tool was used for study design, selection of statistical methods, or interpretation of results. We further reviewed, tested, and validated all AI-suggested code and reviewed and revised all AI-assisted text.
