# Supplementary material for: Compound food failure on a deteriorated mast system drives Japan’s bear conflict crisis
Source: Sci Adv. 2026 Sep 23;12(39):eaej8134. doi: 10.1126/sciadv.aej8134 (PMC13600147; doi:10.1126/sciadv.aej8134)
Supplement: Supplementary file 1 [file sciadv.aej8134_sm.pdf]

Science Advances
AAAS

Supplementary Materials for

Compound food failure on a deteriorated mast system drives Japan's bear conflict crisis

Hengjun Xiao *et al.*

Corresponding author: Tomohiro Ichinose, tomohiro@keio.jp

Sci. Adv. **12**, eaej8134 (2026)
DOI: 10.1126/sciadv.aej8134

This PDF file includes:

Supplementary Text S1 and S2
Figs. S1 and S2
Tables S1 to S8

Supplementary Text

Text S1. Derivation of intrinsic growth rate from Tochigi et al. (*31*) demographic parameters. Assumptions: (i) sex ratio at birth is 1:1; (ii) adult survival is independent of age (no senescence); (iii) all reproductive-age females breed at a constant interval of 2.38 years (the reported mean); (iv) first-year cub survival is approximated by the reported six-month survival; (v) the human-caused mortality rate (q_h = 0.005 / year) reflects low-pressure central-Honshu study area, and the authors themselves caution against extrapolating it to other regions; the estimate derived here accordingly applies only to the Echigo–Mikuni population. Cumulatively, assumptions (iv) and (v) bias the resulting λ upward, the solved value should therefore be interpreted as an upper bound on the intrinsic growth rate consistent with currently available peer-reviewed parameters, rather than as a central estimate.

Parameter	Value	Definition
α	5.44	Age at first reproduction (years), rounded to 5 for discrete summation
Δt	2.38	Reproductive interval (years)
F	1.58	Litter size (cubs)
q_c	0.235	Cub mortality (first six months)
q_n	0.108	Annual natural mortality, subadult and adult
q_h	0.005	Annual human-caused mortality, subadult and adult
ξ	0.5	Sex ratio at birth (assumed)
ω	20	Maximum age observed

The Euler–Lotka equation relates the life-table quantities $l(x)$ (probability of survival from birth to age x) and $m(x)$ (expected number of daughters produced at age x) to the population growth rate λ:

$$\sum_{\alpha}^{\omega} l(x) \cdot m(x) \cdot \lambda^{-x} = 1 \quad (1)$$

Assuming constant adult survival $1 - q_n - q_h$ and constant reproduction at all ages $x \geq \alpha$, $l(x) = (1 - q_c) \cdot (1 - q_n - q_h)^{x-1} = 0.765 \times 0.887^{x-1}$ and $m(x) = F \cdot \xi / \Delta t = 0.332$, so equation (1) simplifies to:

$$\sum_{x=5}^{20} 0.765 \times 0.887^{x-1} \cdot 0.332 \cdot \lambda^{-x} = 1 \quad (2)$$

Solve the equation, $\lambda \approx 1.018$ (intrinsic growth rate $r \approx 1.8\%$ per year).

Text S2. A rectifier model of mast deterioration under cue-threshold crossing. The mechanism rests on three established physiological facts.

1. **Bottleneck.** The multi-year resource bottleneck on conversion from flowers to mature mast in *Fagus crenata* is nitrogen, not carbon. Nitrogen reserves are depleted by mast events and refill slowly over multiple subsequent years (*13*), its accumulation is governed by physiology and soil mineralization rates, neither of which is appreciably accelerated by warming, and the maximum output per cued year is therefore fixed by physiology and cannot easily rise.
2. **Cues.** Flower-bud differentiation is gated jointly by two cues: the plant's current resource state, and post-solstice summer temperature in a cue window that opens abruptly at the solstice (*53-55*).
3. **Rectifier.** The two cues together act as a rectifier on resource use. When either reserves or

summer temperature falls below threshold, reproductive commitment is vetoed and nitrogen is conserved. Historically, periodic cool summers reset reserves and produced the boom-bust mast cycle. After 2006, the temperature arm of this rectifier may have failed: the plant can no longer veto induction during low-reserve years, and the model below shows the predicted consequence to be a descending floor with the ceiling preserved.

Let $N_t \in [0, N_{\max}]$ denote the nitrogen reserve at year t, and T_t the post-solstice mean temperature, drawn from a stationary distribution. The system is

$$\Phi_t = \mathbb{1}(T_t > \tau), \tag{3}$$

$$M_t = \Phi_t \cdot \beta\alpha \cdot \frac{N_t}{N_t + K_N} + (1 - \Phi_t) \cdot M_{\text{base}}, \tag{4}$$

$$N_{t+1} = \min(N_{\max}, \max(0, N_t + r - cM_t)), \tag{5}$$

where τ is the cue threshold; α is the flowering effort in cued years; β, K_N is the conversion peak and half-saturation with nitrogen; M_{base} is the baseline mast in cool-cued years; r is the annual nitrogen replenishment (mean is $\bar{r}$); c is the cost per unit mast. Two regimes follow from the temperature distribution: Regime A ($P(T_t > \tau) = p < 1$), in which cool-cued years let $N_t \to N_{\max}$; and Regime B ($P(T_t > \tau) \to 1$), in which cool-cued years are rare.

Ceiling. The maximum mast output in any year is bounded by

$$M_{\text{top}} = \beta\alpha \cdot \frac{N_{\max}}{N_{\max} + K_N}, \tag{6}$$

which depends only on physiological parameters. It is reached in both regimes whenever $\Phi_t = 1$ and $N_t \to N_{\max}$, guaranteed in A by cool-year recovery, stochastic in B but still occurring in sufficiently long windows.

Floor. Under Regime A, cool-cued years yield $M_t = M_{\text{base}}$. Under Regime B, the system settles around a nitrogen-stationary state determined by mass balance,

$$\bar{r} = c\beta\alpha \cdot \frac{N_B^*}{N_B^* + K_N} \quad \Leftrightarrow \quad N_B^* = \frac{\bar{r}\, K_N}{c\beta\alpha - \bar{r}}, \tag{7}$$

with stochastic drains driving N_t below $N_B{}^*$, during which $M_t \to \beta\alpha N_t / K_N \to 0$. The Regime B floor lies below M_{base} whenever

$$\beta\alpha \cdot \frac{N_B^*}{N_B^* + K_N} < M_{\text{base}}, \text{i.e.}, \bar{r} < c\, M_{\text{base}}. \tag{8}$$

Mean. Stationary mass balance gives $\mathbb{E}[M_t \mid B] = \bar{r} / c$, while

$$\mathbb{E}[M_t \mid A] = (1 - p)\, M_{\text{base}} + p\beta\alpha \cdot \mathbb{E}\left[\frac{N_t}{N_t + K_N} \mid \Phi_t = 1\right], \tag{9}$$

which exceeds $\bar{r} / c$ when cool-year recovery brings N_t near $N_{\max}$.

Numerical illustration. With $\alpha = 1$, $\beta = 5$, $K_N = 0.8$, $N_{\max} = 10$, $\bar{r} = 1.2$, $\sigma_r = 0.20$, $c = 1$, $M_{\text{base}} = 1$, $\tau = 18.5$°C, $\sigma_T = 1.5$°C, and $\bar{T}_A = 18.0$°C, $\bar{T}_B = 20.0$°C — giving $P(\Phi = 1) = 0.37$ and 0.84 respectively, comparable in qualitative magnitude with the 19 % → 45 % shift in high-cue years across the 2006 break. Pooled over 200 stochastic replicates, the median 4-yr rolling min shifts from 1.00(= M_{base}) under A to 0.00under B, while the median 4-yr rolling max shifts from 4.10 to 3.19. The floor traverses its full range; the ceiling moves only 22 % toward zero (Fig. S2).

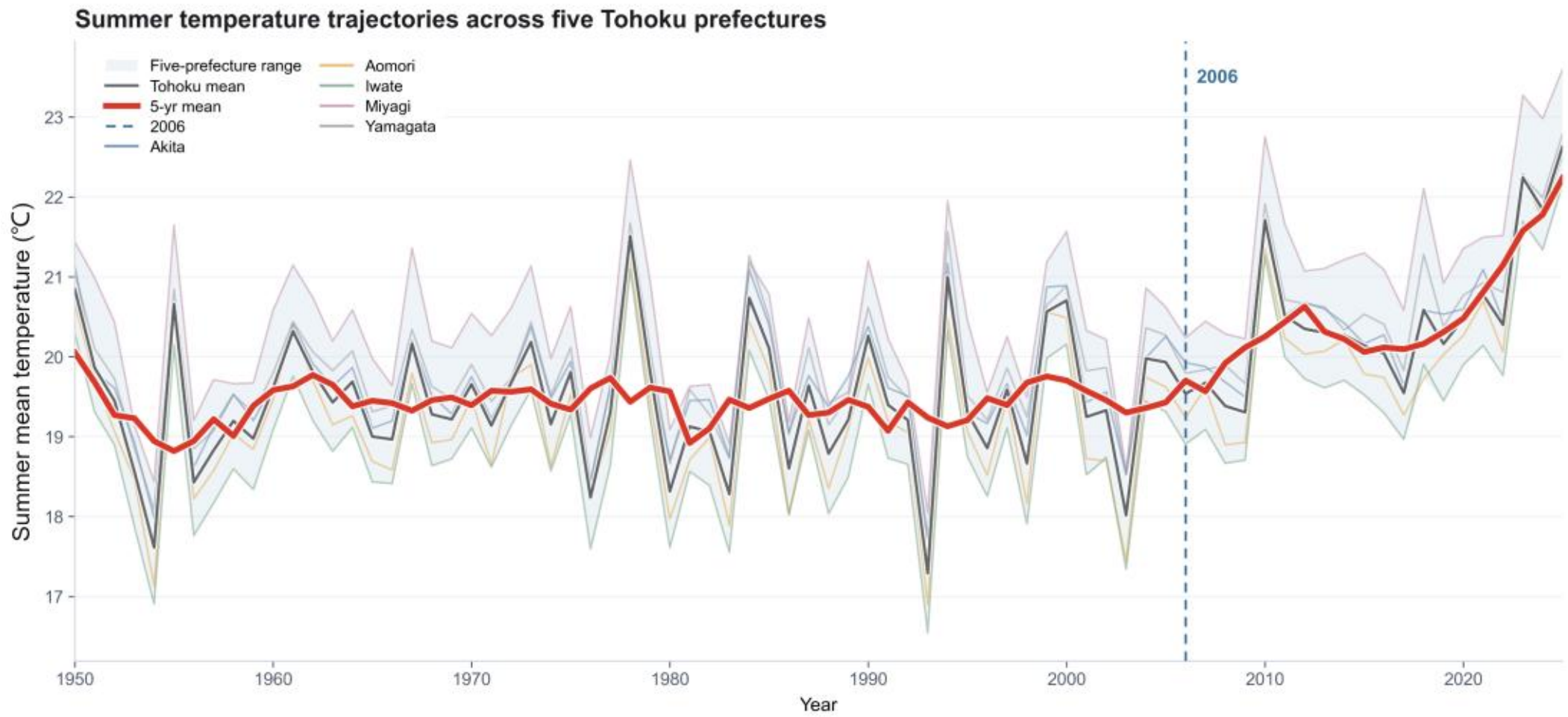

Fig. S1. Summer temperature trajectories in the five Tohoku prefectures, 1950-2025. Thin colored lines show prefecture-specific June-August mean temperature, the shaded band shows the annual range across the five prefectures, the black line shows the Tohoku regional mean, and the red line shows its centered 5-year rolling mean. The dashed vertical line marks 2006.

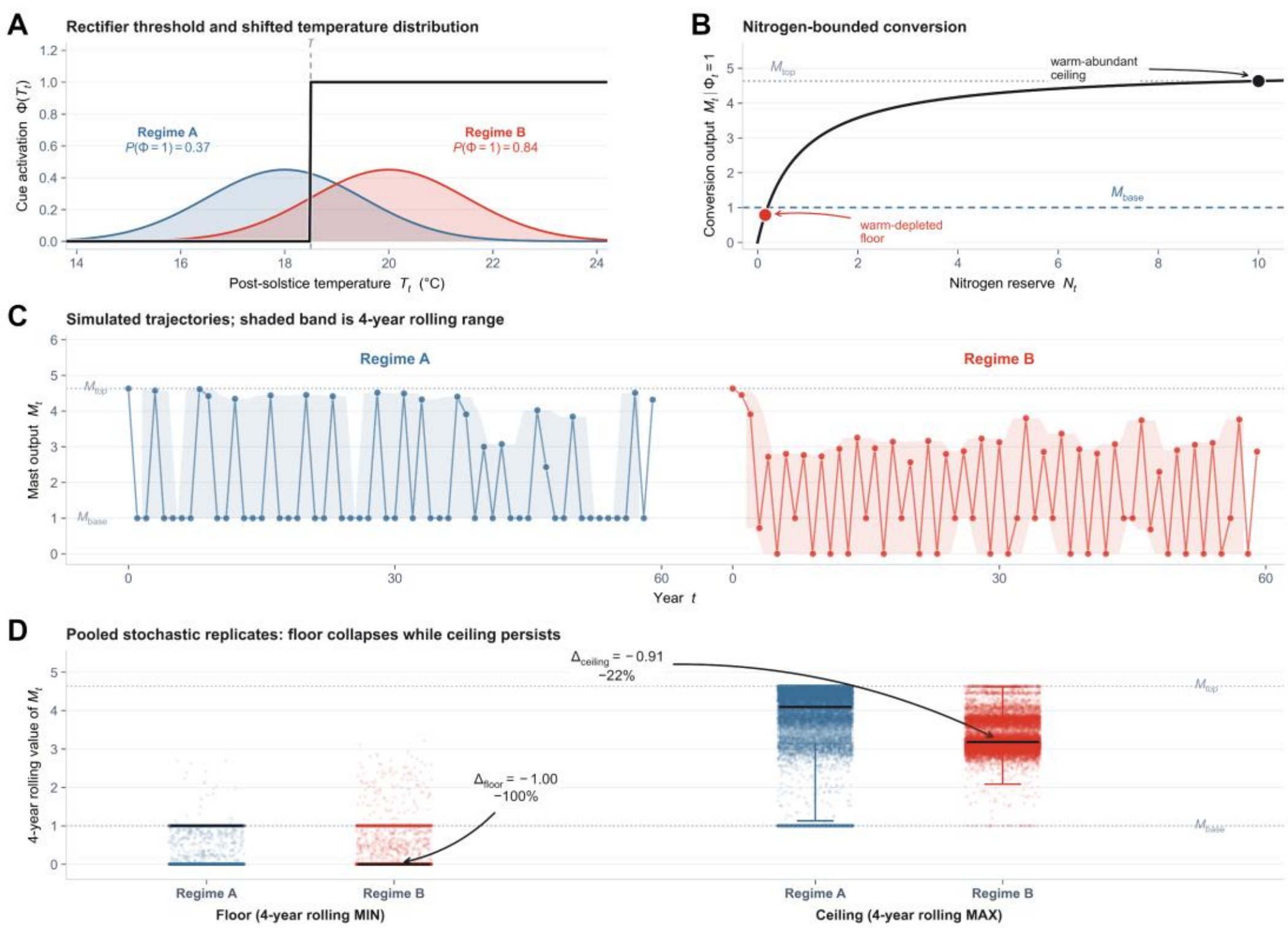

Fig. S2. Rectifier function (black step) and post-solstice temperature distributions. (A) Regime A (blue) straddles the threshold τ with P(Φ = 1) = 0.37; Regime B (red) lies almost entirely above it with P(Φ = 1) = 0.84. (B) Conversion output as a function of nitrogen reserve. Three states are marked: warm-abundant (black; ceiling in both regimes), warm-depleted (red; Regime B floor), and the cool-cued baseline M_{base} (dashed blue; Regime A floor). (C) Simulated 60-year mast trajectories; shaded band is the 4-year rolling envelope. Regime A alternates

binarily between cool-cued and warm-abundant years; Regime B loses this binary structure. (D) Pooled 4-year rolling min (floor) and max (ceiling) across 200 stochastic replicates of 80 years. Floor median: 1.00→0.00 (−100%); ceiling median: 4.10 → 3.19 (−22%).

Table S1. Rolling-window robustness of mast- and flower-system floor and ceiling trends, 1990-2025. Block-bootstrap Mann-Kendall trend tests (2000 replicates, block length = window length) on rolling minima and maxima for both 3-year and 4-year windows, balanced three-prefecture panel.

Series	3-year β/yr	3-year *P*	4-year β/yr	4-year *P*
$MI_{minimum}$ (floor)	−0.0112	0.011	−0.0118	0.004
$MI_{maximum}$ (ceiling)	−0.0187	0.720	−0.0283	0.479
$FI_{minimum}$ (floor)	+0.0046	0.971	+0.0043	0.660
$FI_{maximum}$ (ceiling)	−0.0014	0.497	−0.0091	0.126
$MI_{minimum}$ -$FI_{minimum}$ (difference)	−0.0157	< 0.001	−0.0160	< 0.001
$\mathrm{MI}_{minimum}$ / $\mathrm{FI}_{minimum}$ (ratio)	−0.0176	< 0.001	−0.0179	< 0.001

Table S2. Top five candidate break years from the Sup-Wald level-shift search on the flower-to-mast conversion residual series. Tests within-prefecture demeaned conversion residuals on the balanced three-prefecture panel (Aomori, Iwate, Miyagi) over 26 candidate years (1995-2020) with 15% trimming. Andrew's size-corrected critical values (k = 1, 15% trim) are 7.17, 8.85 and 12.35 at α = 0.10, 0.05 and 0.01 respectively.

Year	F-statistic	Nominal *P*	Shift coefficient
2004	11.17	1.16×10^{-3}	−0.392
2005	14.73	2.15×10^{-4}	−0.438
2006	16.11	1.14×10^{-4}	−0.453
2007	15.40	1.57×10^{-4}	−0.443
2008	10.41	1.68×10^{-3}	−0.372

Table S3. Mechanism-probe model coefficients on the balanced three-prefecture panel versus the full five-prefecture panel. Model: $MI \sim FI$ × Post + MI_{lag1} × Post, with prefecture random intercepts; Post = 1 from 2006 onward. The balanced subset (Aomori, Iwate, Miyagi; n = 105 prefecture-years) has 14 pre-2006 observations per prefecture; the full five-prefecture panel (n = 167) includes Akita and Yamagata records beginning 2001-2004, contributing only roughly 5 pre-break observations each, which dilutes the lag-1 interaction. The balanced panel is therefore the definitive test of the lagged-cost channel; the full-panel column confirms that the within-year per-pulse channel (*FI* × Post) is robust to panel composition while the lagged channel is identifiable only with adequate pre-2006 baseline. Only signs and significance shown for the full-panel column, as the balanced subset is the definitive mechanistic specification.

Term	Balanced β	Balanced *P*	Full β	Full *P*

Post (intercept shift)	−0.071	0.407	—	—
FI	+0.948	< 0.0001	—	—
FI × Post	−0.145	0.0002	(significant)	< 0.0001
MI_{lag1}	−0.111	0.0044	—	—
MI_{lag1} × Post	−0.057	< 0.0001	(n.s.)	0.281

Table S4. Mediation decomposition of the lag-1 reproductive-cost effect on flower-to-mast conversion, pre- vs. post-2006. Path coefficients are estimated by mixed-effects regression with prefecture random intercepts, separately in each regime, on the balanced three-prefecture panel (Aomori, Iwate, Miyagi; n_{pre} = 48, n_{post} = 57 prefecture-years). Confidence intervals and p-values are from within-prefecture moving-block bootstrap (block length = 4 years, 1000 replicates, all converged). Δ denotes the post-minus-pre contrast.

Path	Definition	Pre-2006 [95% CI]	Post-2006 [95% CI]	Δ (post − pre) [95% CI]	*P* (Δ)
a	$MI_{lag1} \rightarrow FI$	−0.425 [−0.546, −0.292]	−0.551 [−0.728, −0.382]	−0.126 [−0.357, +0.097]	0.200
b	$FI \rightarrow MI \mid MI_{lag1}$	+0.951 [+0.885, +1.080]	+0.786 [+0.573, +1.059]	−0.165 [−0.413, +0.105]	0.212
c'	$MI_{lag1} \rightarrow MI$	−0.101 [−0.142, +0.003]	−0.090 [−0.288, +0.087]	+0.011 [−0.228, +0.166]	0.946
a×*b*	Indirect effect	−0.404 [−0.532, −0.282]	−0.433 [−0.623, −0.262]	−0.029 [−0.255, +0.178]	0.702
c'+*a*×*b*	Total lag1 effect	−0.506 [−0.607, −0.357]	−0.523 [−0.726, −0.346]	−0.017 [−0.280, +0.176]	0.634

Mediated share ($a \times b$ / total): pre-2006 = 80.0%; post-2006 = 82.8%. Decomposition of $\Delta(a \times b)$ = −0.029 into its three sources, include($\Delta(a)$= −0.120, $\Delta(b)$= +0.070 and cross term $\Delta(a) \times \Delta(b)$= +0.021)

Table S5. Six candidate NB-GLMMs for prefecture year incident counts (n = 90; five Tohoku prefectures, 2008–2025) compared by AIC. All models include a prefecture random intercept and an NB dispersion term. Fixed effect covariates are mast deficit severity *Sev* = max(0, 0.38 - *MI*), the detrended annual ($PsnNet_d$), and the mean centered year ($Year_c$). Model M_e is selected for subsequent attribution and trigger analyses. *k* denotes the number of fixed effect terms (excluding the intercept); all models additionally estimate the prefecture intercept variance and NB dispersion.

Model	Fixed effects	*k*	AIC	ΔAIC	w_{AIC}
M_a	$Sev + Year_c$	2	488.7	14.2	<0.001
M_b	$Sev + PsnNet_d$	2	481.8	7.3	0.012
M_c	$Sev + Year_c + PsnNet_d$	3	477.0	2.5	0.132
M_d	$Sev \times Year_c + PsnNet_d$	4	476.3	1.8	0.180
M_e	$Sev \times PsnNet_d + Year_c$	4	474.5	0.0	0.455
M_f	$Sev \times Year_c + Sev \times PsnNet_d$	5	475.9	1.4	0.221

Table S6. Sensitivity of regime-shift attribution and $Sev \times PsnNet_d$ interaction across the mast-failure threshold envelope. Counterfactual attribution and the M_e model were refits at five thresholds spanning the ΔAIC < 2 envelope (*MI* ∈ [0.22, 0.58]). Attribution stayed within 12–

16%, and the interaction remained negative throughout (β from −0.025 to −0.012). β(*Sev* × $PsnNet_d$) varies modestly with threshold because Sev = max(MI_t − *MI*, 0) is re-defined at each cut-point; its sign and the qualitative conclusion are unchanged.

Threshold	Crisis prefecture-years	Counterfactual crisis	Prevented incidents	% of expected	β	*P*
0.22	23	9	118	15.0%	-0.025	0.209
0.30	23	15	126	16.1%	-0.025	0.055
0.38	26	18	120	15.3%	-0.020	0.034
0.46	29	21	110	14.1%	-0.016	0.033
0.58	31	25	96	12.2%	-0.012	0.039

Table S7. Out-of-sample prediction of outbreak-year incidents under leave-one-year-out refitting. For each outbreak year, M_e and a no-interaction comparator (M_c) were refitted on the panel excluding that year and used to predict prefecture-year incidents in the held-out year. The "Ratio" column is the per-prefecture M_e / no-interaction predicted ratio, values near 1.0 indicate the interaction term is dormant (consistent with $PsnNet_d$ ≈ baseline), values substantially above 1.0 indicate the interaction is active (consistent with synchronous primary production collapse).

Held-out year	Prefecture	Observed	M_e (OOS)	No-int (OOS)	Ratio
2023 (single channel)	Akita	62	21.3	23.3	×0.92
	Aomori	10	8.4	8.6	×0.98
	Iwate	46	31.8	35.6	×0.89
	Miyagi	3	4.9	6.2	×0.79
	Yamagata	5	5.2	5.9	×0.88
	TOTAL	126	71.7	79.6	×0.90
2025 (compound)	Akita	59	109.1	42.0	×2.59
	Aomori	10	15.4	9.2	×1.67
	Iwate	39	82.7	39.7	×2.08
	Miyagi	5	9.8	5.7	×1.73
	Yamagata	12	33.5	10.4	×3.22
	TOTAL	125	250.5	107.0	×2.34

Table S8. Robustness of the *Sev* × $PsnNet_d$ interaction term to outbreak-year exclusion. Refits of the lowest-AIC specification M_e on the full panel and on nested subpanels excluding the synchronized outbreak years (2023 and 2025). The interaction remained significant after excluding 2025 alone, and although significance was lost after excluding both outbreak years, the effect size was largely preserved (β = -0.0238 vs. -0.0203 in the full panel; 17.0% shift), indicating a power-limited rather than outbreak-year-dependent loss of support.

Panel	*n*	β	SE	*P*	ΔAIC vs non-interaction
Full panel	90	−0.020	0.010	0.034	+2.48
Drop 2023	85	−0.023	0.008	0.005	+5.14

Drop 2025	85	−0.044	0.016	0.006	+5.00
Drop 2023 + 2025	80	−0.024	0.017	0.164	−0.04
